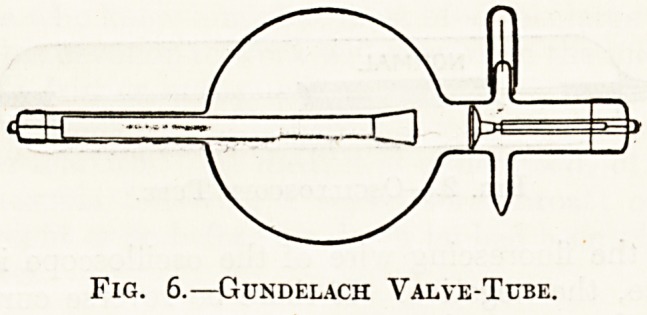# The X-Rays

**Published:** 1912-08-31

**Authors:** Alfred C. Norman

**Affiliations:** House Surgeon at Durham County Eye Infirmary.


### ELECTRICITY IN MODERN MEDICINE.*

### XVII.—The X-Rays.

By ALFRED C. NORMAN, M.D. Edin., House Surgeon at Durham County Eye Infirmary.

Fig. 1 illustrates the general outline of an oscilloscope tube at rest.

Fig. 2 shows diagrammatically how such a tube may appear when excited by an induction coil. There is fluorescence (indicated by the shaded lines) round one wire only, hence we know that the current from the coil is practically unidirectional.

Fig. 3 also illustrates an oscilloscope tube excited by an induction coil, but in this case there is fluores-

Fig. 1.—Oscilloscope Tube.

cence round both wires—a sure indication that the coil is generating reverse current. The proportion of reverse to that of normal current may be roughly estimated by observing how far the fluorescence extends along each wire.

An oscilloscope tube also serves as a pole indicator for the induction coil. If there be no reverse current the terminal of the coil which is connected

Fig. 2.—Oscilloscope Tube.

with the fluorescing wire of the oscilloscope is, of course, the negative. If there be reverse current, then the terminal connected with the wire of the oscilloscope which shows *most* fluorescence must be regarded as negative. Of course, in the latter case both terminals are really negative and positive alternately many thousands of times per second, but we ignore the current at "make" (*i.e.*, the reverse current) and name the terminals in accordance with

Fig. 3.—Oscilloscope Tube.

their polarity to the break discharges—*i.e.*, the direct current. (See page 63.)

### Milliampere-Meter.

Fig. 4 illustrates an excellent type of d'Arsonval milliampere-meter designed for *x*-ray work.

It will be noticed in this particular instrument that zero is in the centre of the scale and that the needle is free to travel either to the right or to the left; the advantage of this being that the meter also serves to indicate the direction in which the current is flowing. For instance, if the needle move towards the right we know that current is flowing through the meter from the terminal on the left (A) towards the terminal on the right (B), and this indicates the direction of the current in the whole circuit and incidentally tells us which pillar of the induction coil is positive and which negative.

A word of warning must here be given. A milliampere-meter should never be connected directly with the two pillars of an induction coil in order to test the polarity of the latter; for the meter has practically no resistance and this procedure would be tantamount to short-circuiting the coil through the meter with disastrous results to the delicate mechanism of the meter. A suitable resistance, such as an *x*-ray tube or an air-gap of at least eight inches, must always be interposed in the secondary

Fig. 4.—Milliampere-Meter, by Schall and Son.

circuit, in series with the meter, in order to protect it from the violence of the coil.

### Reverse Current, Spark-Gap, Valve-Tubes.

We have already seen that reverse current is that current which is produced at each "make" of the primary circuit; that it cannot be utilised in the satisfactory production of *x*-rays; that it is less in amount and of lower voltage than the "make" current; that it tends to spoil tubes and blur the outline of radiograms; and, finally, that its presence renders unreliable the reading of the milliamperemeter.

In the section on induction coils it was pointed out that by keeping the self-induction of the coil *high* and the voltage of the primary circuit *low* we

* Previous articles appeared on Nov. 11, 25, Dec. 9, 30, Jan. 13, 27, Feb. 17, March 9, 30, April 20, May 4, 25, June 8, July 6, and Aug. 3, 17.

could considerably reduce the amount of reverse current. It now remains for us to describe some extrinsic devices which can be utilised to suppress this undesirable current.

The simplest of these is the *spark-gap*. It depends upon the fact that the voltage of the reverse current is considerably less than that of the direct current; hence, by interposing a suitable resistance in the secondary circuit, it is possible, without markedly retarding the flow of the direct current, to prevent any reverse current from passing. This could be very easily done by dividing one of the wires leading to the $x$-ray tube and separating the cut ends so as to make the current spark across a gap of, say, 1½ inch before reaching the tube. The resistance of this little air-gap would be sufficient to suppress a good deal of reverse current, but in practice it is customary to take advantage of another property of high tension electricity which greatly adds to the efficiency of the spark-gap. It is found

Fig. 5.—Variable Spark-Gap, showing proper connection to the cathodal pillar of the Induction Coil.

that current will spark from a sharp point to a flat metal plate very readily *if the point be positive*, but that the resistance of the air-gap becomes very high if the current be reversed so as to make the plate positive and the point negative. By arranging a spark-gap, therefore, so that the direct current has to jump from a point to a plate the latter will be hardly retarded at all, but we shall be interposing a tremendous resistance in the path of the reverse current, for it will have to jump from a plate to a point; hence a very much smaller gap will serve to suppress the reverse current.

A spark-gap makes a good deal of noise in working, so it is usually enclosed in a glass jar to deaden this as much as possible. Fig. 5 illustrates the usual pattern of spark-gap and the way in which it must be connected with the induction coil—namely, with *the plate of the spark-gap to the negative pillar of the coil*. The point is adjustable by screwing the rod R and should be just sufficiently far from the plate to suppress the reverse current and no farther. The oscilloscope tube will, of course, indicate when the proper distance has been arrived at.

The *valve-tube* is similar in principle to the spark-gap but it makes no noise and is rather more efficient. We have seen that a fairly hard $x$-ray tube acts to a considerable extent as its own valve, simply because it has sufficient resistance to suppress reverse current but not sufficient to retard direct current. The exhaustion of a valve-tube and its resistance to direct current are considerably less than is the case in an ordinary $x$-ray tube, but its electrodes are so shaped that they afford a maximum of resistance to the passage of reverse current; it is, therefore, absolutely essential that the valve-tube be properly connected to the induction coil. The electrode which presents the largest surface of metal inside the tube must be connected with the cathode of the coil, but as a general rule the makers mark the tubes as they should be connected.

Valve-tubes should be fitted with an "Osmo" regulator so that their vacuum can be reduced from time to time, otherwise they would gradually become so hard as to retard even the passage of direct current. Their resistance should be less than half-inch equivalent spark to direct current and between two and four inch equivalent spark to reverse current.

Fig. 6 illustrates a Gundelach valve-tube with "Osmo" regulation.

Another device for suppressing reverse current is the "*mica-valve*" introduced by Mr. Leslie Miller. This consists of a series of mica discs rotating synchronously with the interrupter and so timed that they intercept the secondary current during the

Fig. 6.—Gundelach Valve-Tube.

"make" phrase. The writer has had no experience of this ingenious device, but it is said to be quite satisfactory.

### The Switch-Board.

It is customary to mount on a marble slab or "switch-board" the various accessories for controlling and measuring the current in the primary circuit and to fix this on a convenient wall in the $x$-ray room.

There should be two separate and distinct circuits on the switch-board—one for driving the motor which rotates the interrupter; the other for regulating the current which passes through the primary circuit of the coil.

The *motor circuit* consists of two terminals for connecting with the motor, a switch for starting the latter, and a small sliding rheostat for controlling its speed. These, including the rheostat, are all in series.

The *coil circuit* should consist of a pair of terminals for connecting with the primary of the induction coil, an ampere-meter and a volt-meter, a knife switch, and a very powerful resistance for regulating the amount of current passing through the primary of the coil.

(*To be continued.*)

## Figures and Tables

**Fig. 1. f1:**
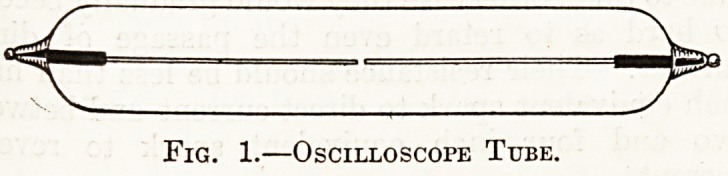


**Fig. 2. f2:**
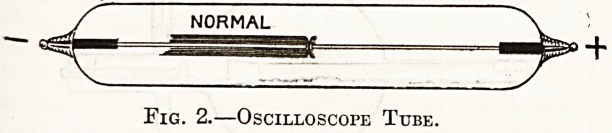


**Fig. 3. f3:**
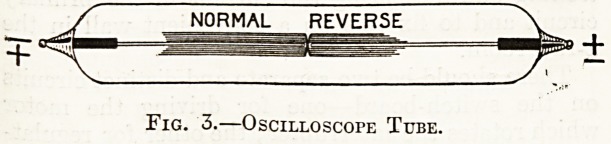


**Fig. 4. f4:**
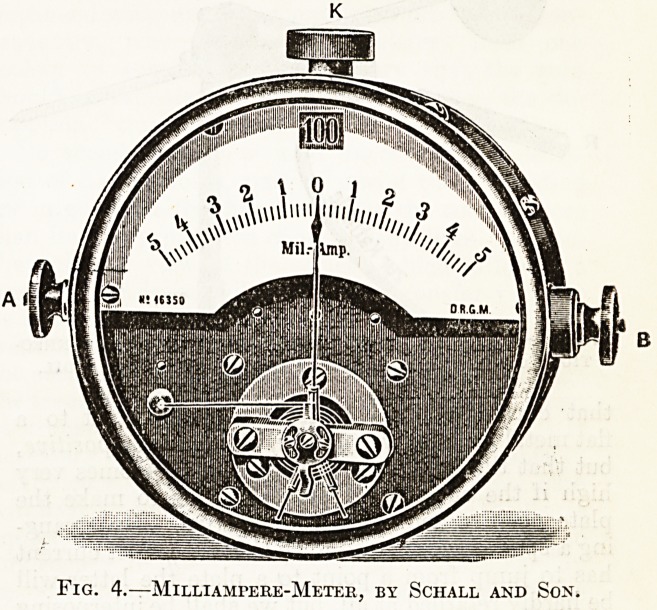


**Fig. 5. f5:**
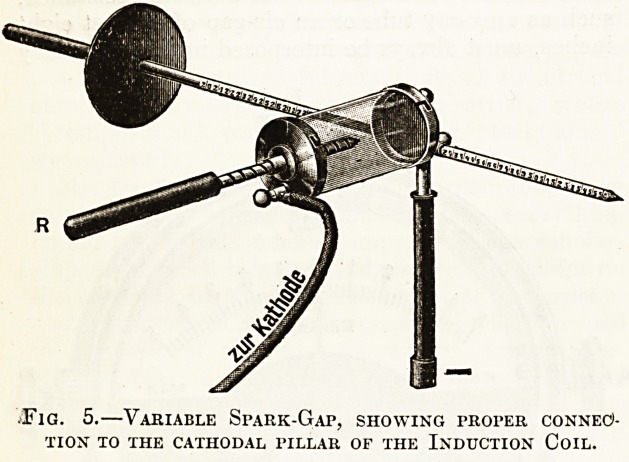


**Fig. 6. f6:**